# Development and application of the active surveillance of pathogens microarray to monitor bacterial gene flux

**DOI:** 10.1186/1471-2180-8-177

**Published:** 2008-10-09

**Authors:** Richard A Stabler, Lisa F Dawson, Petra CF Oyston, Richard W Titball, Jim Wade, Jason Hinds, Adam A Witney, Brendan W Wren

**Affiliations:** 1 Department of Infectious and Tropical Diseases, Keppel Street, London School of Hygiene and Tropical Medicine, London, WC1E 7HT, UK; 2 Biomedical Sciences, DSTL, Porton Down, Salisbury, SP4 0JQ, UK; 3 School of Biosciences, The University of Exeter, The Queen's Drive, Exeter, Devon, EX5 5QJ, UK; 4 King's College Hospital, Denmark Hill, London, SE5 9RS, UK; 5 Bacterial microarray group, St. George's, University of London, Cranmer Terrace, London, SW17 0RE, UK

## Abstract

**Background:**

Human and animal health is constantly under threat by emerging pathogens that have recently acquired genetic determinants that enhance their survival, transmissibility and virulence. We describe the construction and development of an Active Surveillance of Pathogens (ASP) oligonucleotide microarray, designed to 'actively survey' the genome of a given bacterial pathogen for virulence-associated genes.

**Results:**

The microarray consists of 4958 reporters from 151 bacterial species and include genes for the identification of individual bacterial species as well as mobile genetic elements (transposons, plasmid and phage), virulence genes and antibiotic resistance genes. The ASP microarray was validated with nineteen bacterial pathogens species, including *Francisella tularensis*, *Clostridium difficile*, *Staphylococcus aureus*, *Enterococcus faecium *and *Stenotrophomonas maltophilia*. The ASP microarray identified these bacteria, and provided information on potential antibiotic resistance (eg sufamethoxazole resistance and sulfonamide resistance) and virulence determinants including genes likely to be acquired by horizontal gene transfer (e.g. an alpha-haemolysin).

**Conclusion:**

The ASP microarray has potential in the clinic as a diagnostic tool, as a research tool for both known and emerging pathogens, and as an early warning system for pathogenic bacteria that have been recently modified either naturally or deliberately.

## Background

Despite advances in the treatment of infectious disease, pathogenic bacteria represent one of the most important threats to health worldwide. Many infectious disease agents have never been controlled or have re-emerged as global pathogens, while others pose a new threat. Recently emerged pathogens of heightened virulence include *Vibrio cholerae *O139 strains [1,2], *Escherichia coli *0157 [3,4], *Salmonella enteritidis *phage type-4 [5] and the multidrug resistant phage type-DT104, all of which have emerged over the last decade as a global health problem in both human and animal disease [6]. There is now clear evidence that these pathogens have become more virulent by acquiring genome segments through lateral gene transfer that result in gain-of-function traits [7]. This is dramatically illustrated by the transfer of antimicrobial resistance determinant among pathogenic (and non-pathogenic) bacteria such as meticillin resistant *Staphylococcus aureus *(MRSA) and multidrug resistant enterococci. The identification of newly acquired potentially harmful genetic elements and antibiotic resistances could influence clinical practice and policy, resulting in more effective treatment. Additionally, this identification of new traits could prove vital in the identification of bioterrorism threats, where the deliberate release of highly virulent pathogens is a major concern, particularly if the genome of the infectious agent has been modified with virulence determinants for nefarious purposes.

In the last few years, research in microbial pathogenesis and molecular epidemiology has changed fundamentally, from a piecemeal approach of characterising individual determinants or point mutations to a global analysis of pathogen genomes, fuelled by the ability to determine the complete genome sequence of microorganisms. This enabled the development of high-throughput nucleic acid hybridisation technologies including macro- and microarrays involving amplified gene fragments and oligonucleotide arrays (*e.g. *Affymetrix GeneChips). Microarrays have the capacity to rapidly monitor the genome content of bacterial strains and identify horizontal gene transfer elements, using thousands of reporter elements in a given experiment. Horizontal gene transfer is a major evolutionary mechanism for bacteria [8,9], methods that can demonstrate gene acquisition (and loss) are crucial in identifying emerging pathogens, and could act as an early warning of the emergence of a potentially more virulent strain. At present microarray technology is limited to mainly single species microarrays. Yet the continued expansion of available genome sequence data and our increased understanding of the genetic basis of microbial virulence, this has presented a unique opportunity to monitor the genome content of microorganisms and the emergence of more virulent pathogens. With this in mind we report the development and application of an Active Surveillance of Pathogens (ASP) microarray for monitoring gene flux in pathogens, antimicrobial resistance and virulence profile, along with potentially identifying gene acquisitions and new outbreak strains. The ASP array is unique in that it represents known virulence determinants, antibiotic resistance genes and pathogenicity traits from 151 bacteria species, covering a broad range of species and genera on a single microarray. This provides an unparalleled opportunity to study gene flux and identify novel traits. The ASP microarray was validated with 19 bacterial species, ranging from those who's fully curated genome sequences were available, to those that were unsequenced, with little or no background information; including a blind control of a sample from unknown origins. The ASP array identified both known and unknown samples, including; *Francisella tularensis*, *Clostridium difficile, Staphylococcus aureus*, Vancomycin resistant *Enterococcus faecium *(VRE) and *Stenotrophomonas maltophilia *and provided information on drug resistance and the presence of potential virulence determinants.

## Methods

### Selection of oligonucleotide gene reporters for microarray

Prior to the design of the oligonucleotides, a database of potential bacterial coding sequences (CDS) was established using published completed genome sequences; thus ensuring consistency in gene location and identity. A Perl script was used to extract CDS sequences from the sequence reference database (RefSeq) , which consists of curated completed and published genomes. Reporters for the ASP microarray were designed from CDS categorised by TIGR as either 'cell adhesion', 'detoxification', 'toxin production & resistance' or 'pathogenicity'  or TIGR Annotation search . Alongside these, additional reporters were designed based on ribosomal protein subunit genes, these were selected for their species, genus and family specificity. 16S/23S genes were not used due to their high degree of conservation.

The oligonucleotide reporters were designed using OligoArray2.1 software [10]. Irrespective of GC content of the target genome the following parameters were set; GC content 40–60%, Tm 85–90°C and runs of five homologous bases were prohibited. If OligoArray2.1 failed to design oligonucleotides for the majority of CDS, relaxed parameters were applied as follows; maximum GC-content was increased to 68% and maximum Tm increased to 94°C for high GC-content organisms. For low GC-content organisms the minimum GC-content was lowered to 35% and the minimum Tm decreased to 83°C. Oligonucleotides were checked by BLASTN [11] using default settings but with a seed word length of seven (due to short oligonucleotide sequence length) and an e-value cut-off of 0.0001, against all CDS from all available species within a genera. If any oligonucleotides, had a match with a bit score of greater than 72 (approximately >45/50 bp match) to CDS other than that on which it was designed, the oligonucleotides for these CDS were considered redundant and thus removed i.e. one oligonucleotide will produce signal from homologous CDS, this accounts for duplicated genes or gene families. Subsequently a self-self BLAST for all selected oligonucleotides was performed to check for redundancy within the oligonucleotides, again any two oligonucleotides which shared greater than 45/50 base pair identity were deemed to be a duplication and one was removed from the potential oligonucleotide list. This iterative approach allows for expansion of only novel reporter elements with the addition of each newly published genome sequence. The current ASP microarray (v5.3) consists of 4958 oligonucleotide reporters (additional file 1) designed from 99 bacterial genera (151 species, 205 genomes, plus at least 100 mobile genetic elements (MGE) additional file 3).

### Construction of ASP microarrays

All oligonucleotides were synthesised by MWG Biotech (Ebersberg, Germany). Oligonucleotides were suspended in 50% DMSO at a final concentration of 50 μM. Microarrays were constructed as described previously [12] by robotic spotting of oligonucleotides in duplicate on UltraGaps amino-silane coated glass slides (Corning, USA). The microarrays were post-print processed according to the slide manufacturer's instructions.

### DNA extractions

Where strains were provided, they were grown in the recommended media, at the recommended temperature. DNA isolations was performed using either the wizard DNA extraction kit (Promega) or the Puregene DNA isolations kit (Gentra Systems) in accordance with the manufacturer's protocols for extraction of either Gram positive or Gram negative bacteria as appropriate. For *C. difficile*, the Puregene Gram positive extraction method was utilised with the addition of 175 μg lysozyme to aid cell lysis, followed by snap freeze using a dry ice and ethanol bath. Samples were then heated to 80°C for 15 min, and the manufacturer's protocol was then resumed.

### Hybridisation conditions & data acquisition

Test samples were labelled and hybridised as described previously [13] using 3–6 μg DNA with a formamide based hybridisation buffer solution (30% formamide, 3.75× Denhart's solution, 3.75 × SSC, 0.75 mM Na_4_P_2_O_7_, 37.5 mM Tris pH 7.4, 0.075% SDS, 0.056 mg/ml used tRNA, modified from [14]) in a final volume of 48 μl at 50°C for 16–20 hours. The microarrays were washed as described previously but with the initial wash at 50°C [13]. The microarrays were scanned using a 418 microarray Scanner (Affymetrix, USA) and intensity fluorescence data acquired using ImaGene 5.5 (BioDiscovery, USA). After scanning, a commercial SpotCheck kit (Invitrogen) consisting of Cy3 labelled random 9-mers was used to hybridize to the microarray, which binds to all the reporters on the microarray. The microarray was then washed and scanned as before but without preheating the initial wash. This step is essential due to the small number of reporters, which hybridise with specific samples and serves as a valuable control to confirm microarray reporter presence on the microarray.

### Data analysis

**Figure 1:**
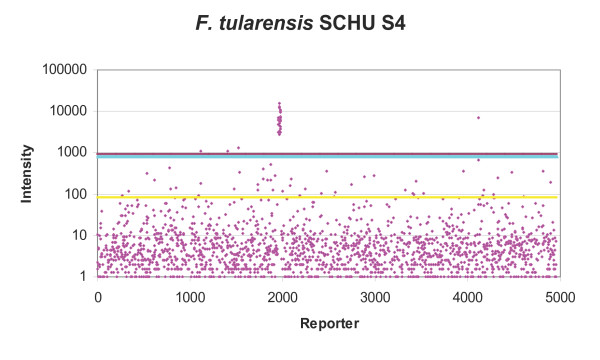
**Distribution of ASP reporter intensities and positive signal selection cut-offs for *F. tularensis *SCHU S4**. Pink dots = Individual ASP reporters (mean) signal, yellow line = mean fluorescence of all ASP microarray reporters, light blue line = one standard deviation (SD) of all ASP microarray reporters, mauve line = mean + SD, reporters with signal greater than mean+SD were selected as positive.

**Figure 2:**
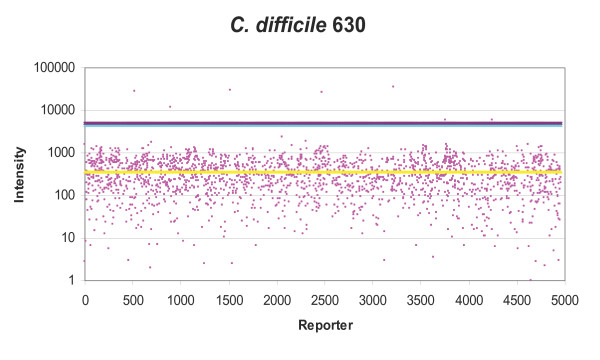
**Distribution of ASP reporter intensities and positive signal selection cut-offs for *C. difficile *630**. Pink dots = Individual ASP reporters (mean) signal, yellow line = mean fluorescence of all ASP microarray reporters, light blue line = one standard deviation (SD) of all ASP microarray reporters, mauve line = mean + SD, reporters with signal greater than mean+SD were selected as positive.

**Figure 3:**
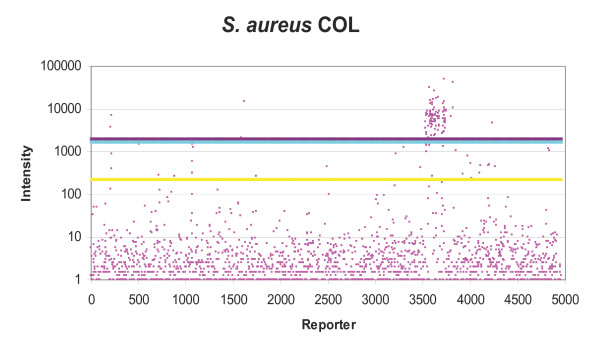
**Distribution of ASP reporter intensities and positive signal selection cut-offs for *S. aureus *COL**. Pink dots = Individual ASP reporters (mean) signal, yellow line = mean fluorescence of all ASP microarray reporters, light blue line = one standard deviation (SD) of all ASP microarray reporters, mauve line = mean + SD, reporters with signal greater than mean+SD were selected as positive.

Initially positive reporter identification was attempted using the proprietary algorithms of ImaGene and BlueFuse software however these lacked the specificity and defined cut-offs required. The method described by Cassone *et al*. [15] was used to identify positive reporters. Each reporter was present on the ASP microarray in duplicate, for each replicate the median of the background signal was deducted from the reporter spot median fluorescence using Excel to give hybridisation signal (figure 1, 2, 3). The mean (m) and standard deviation (sd) of replicate hybridisation signals was calculated. Additionally the background corrected mean (M) and standard deviation (SD) for the whole microarray was calculated (figure 1, 2, 3). The data analysis methodology utilised two main calculations to determine whether a particular reporter was present in any given strain. Firstly, for true positives the signal should be consistent between microarray replicates, therefore if the signal mean was greater than the standard deviation of the spot intensities for a particular reporter (m>sd) this was given a pass, whereas sd>m was given a fail. Secondly, most of the reporters on the microarray do not produce signal, therefore a true positive reporter must be significantly brighter than the background reporters, therefore if the reporter signal mean (m) was greater than the microarray mean M plus SD (m>M+SD) this was given a pass and m<M+SD a fail (figure 1, 2, 3). Reporters were considered present if given a pass for both tests. The resultant gene lists were then entered into GeneSpring. Subsequently in GeneSpring the list of positive reporters was compared to a database of lists consisting of: BLAST predictions, results from previous hybridisations and lists of all the oligonucleotides from each species present in the design database. This identified similarities between gene lists and hybridisation results. For each reporter list, the hypergeometric probability (p-value) was calculated. This probability indicates the level of similarity between gene lists, whereby the p-value was the probability that two gene lists (n & m) share a subset of genes (k) if randomly selected from all ASP microarray genes (u). A low p-value indicates that the lists were not similar by chance.

$$\frac{1}{\left(\begin{array}{c} u\\ m \end{array}\right)}\sum_{i=k}^{n}\left(\begin{array}{c} m\\ i \end{array}\right)\left(\begin{array}{c} u-m\\ n-i \end{array}\right)$$

The test gene list was then added to the database. The continual addition to this database enables comparison to be made between the database and future samples to predict gene content and some degree of identification, for both known and unknown samples.

### Vectorette PCR

Vectorette PCR [16] enables the amplification of specific DNA fragments where only one primer, in this case a 50 mer reporter, is known and was employed to validate the hybridisations. Genomic DNA was digested with either, *Alu*I (Promega), *Eco*RV (Promega), *Pvu*II (Promega) or *Rsa*I (NEB) and heat-inactivated where appropriate, in accordance with the manufacturer's protocol. The vectorette bubble unit was prepared by annealing the oligonucleotides LDF1F and LDF1R (additional file 2). The vectorette unit was then ligated to the individual digested DNA, using T4 ligase (Promega) in accordance with the manufacturer's protocol. Nested 30–32 mer oligonucleotides were designed from within the 50 mer microarray reporter (additional file 2), along with nested primers complimentary to the vectorette bubble (additional file 2). Primary PCR reactions were performed using the vectorette internal primer LDF2, and the gene specific primer (5 μM of each primer). The PCR reactions were performed using 2.5 units of *Taq *polymerase (Promega) in accordance with the manufacturer's protocol, 35 cycles of 92°C 30 sec, 38°C 1 min, 72°C 1 min and a final cycle 72°C 10 min. The secondary PCR was performed using the primary PCR reaction, with nested gene specific primer and nested vectorette primer. A total of 40 cycles of 92°C 30 sec, 55°C 1 min and 72°C 1 min with a final cycle 72°C 10 min. The secondary PCR products were separated on an agarose gel and gel extracted using the QiaQuick gel extraction protocol (Qiagen) in accordance with the manufacturer's protocol. The purified PCR products were then cloned into pGEM (Promega) in accordance with the manufacturer's protocol and sequenced, using M13 forward and reverse primers (additional file 2). The resultant sequences were run through BLAST to identify the cloned genes.

### Results

#### Choice of gene reporters for the ASP array

The ASP microarray was designed to function as a single Cy dye microarray, giving present/absent information about genes based on whether a reporter fluoresces or not. This is not a quantitative comparative assay, therefore two samples can be analysed independently on each microarray simultaneously by labelling one sample with Cy3 and the other with Cy5. The concept microarray (ASPv1) was performed to compare oligonucleotides and PCR product microarrays using important pathogenic bacteria including *Mycobacterium tuberculosis*, *S. aureus *and *Y. pestis *(data not shown) and oligonucleotides were selected for use as reporters.

#### Hybridisation of DNA from bacterial pathogens

Validation was carried out using a selection of 19 bacteria (table 1) which fell into three "Rumsfeld" categories; 'known knowns' (KK) – consisting of fully sequenced and curated genome sequences, which enable BLAST predictions to be performed, 'known unknowns' (KU) – bacteria where partial or no sequence information is available, but for which limited information is available regarding species and drug resistances, and lastly 'unknown unknowns' (UU) – unsequenced bacteria with no prior information regarding species or drug resistance profile.

**Table 1:** Bacterial species tested on the ASPv5.3 to date

**Bacterial ** **species**	**Genome ** **available?^1^**	**Rumsfeld ** **Category**	**Ribosomal ** **predictions^7^**	**No. strains tested ** **on ASP microarray**					**Correct ID ** **p-value^2^**	**Best ** **mis-ID^3^**	**mis- ** **ID^3^** **p-value**
							
				**v1**	**v2**	**v3**	**v4.3**	**v5.3**			
*Acinetobacter baumannii *(γ-proteobacteria)	No	UU	no ribosomals		1	1		1	N/A (0+12)^4^	None^5^	-
*Bacillus anthracis *(firmicutes)	Yes	KK	n/a	1	1		3	3	4.43e-170 (0+122)	*B. cereus*	1.31e-133
*Burkholderia mallei *(β-proteobacteria)	Yes	KK	n/a				3	3	1.55e-179 (5+136)	*B. pseudomallei*	1.34e-163
*Burkholderia pseudomallei *(β-proteobacteria)	Yes	KK	n/a				3	3	6.61e-171 (2+159)	*B. mallei*	3.43e-110
*Clostridium difficile *(firmicutes)	Yes	KK	n/a	1	1	9	12	4	1.11e-4 (0+3)	None	-
*Clostridium sordellii *(firmicutes)	No	UU	no ribosomals			1		1	N/A (0+2)	None	-
*Clostridium tetani *(firmicutes)	No	UU	no ribosomals			1		1	N/A (0+1)	None	-
*Coxiella burnetii *(γ-proteobacteria)	Yes	KK	n/a				2	3	3.32e-19 (0+11)	None	-
*Enterobacter sakazakii *(enterobacteriaeae)	No	UU	γ-proteobacteria (10/14 enterobacteriaceae)			14	11	14	N/A (10+13)	*E. coli*	1.35e-11
*Enterobacter cloacae *(enterobacteriaeae)	No	UU	γ-proteobacteria (10/11 enterobacteriaceae)		1	1		1	N/A (3+19)	*E. coli*	3.47e-7
*Entrococcus faecium *(firmicutes)	No	UU	Firmicutes		2	2	2		N/A (1+11)	*S. aureus*	7.77e-4
*Escherichia coli *(γ-proteobacteria)	Yes	KU	n/a	1	1	1		1	8.82e-9 (8+23)	*S. flexneri*	1.35e-4
*Francisella tularensis *(γ-proteobacteria)	Yes	KK	n/a				3	3	7.33e-65 (3+24)	None	-
*Klebsiella pneumoniae *(γ-proteobacteria)	No	UU	n/a		1	1		1	N/A (0+8)	*E. coli*	3.19e-4
*Legionella pneumophilia *(γ-proteobacteria)	Yes	KK	n/a				3	3	7.08e-102 (0+58)	None	-
*Staphylococcus aureus *(firmicutes)	Yes	KK/KU	n/a	2	1	1		3	8.18e-168 (KK) (4+98) 5.87e-129 (KU) (10+102)	None	-
*Stenotrophomonas maltophilia *(γ-proteobacteria)	No	KU^6^	proteobacteria (γ- or β-)		1	1		1	N/A^6 ^(1+6)	None	-
*Yersinia pestis *(γ-proteobacteria)	Yes	KK	n/a	3	2			2	1.35e-219 (11+188)	*Y. pseudoTB*	5.32e-202
*Yersinia pseudotuberculosis *(γ-proteobacteria)	Yes	KK	n/a			3		3	8.65e-199 (10+129)	*Y. pestis*	3.44e-188

1 = Indicates availability of at least one sequenced strain at time of analysis. 2 = example p-value for correct prediction (on ASPv5.3), 3 = example of best incorrect prediction and p-value (on ASPv5.3), 4 = number of positive reporters from example hybridisation (ribosomals + 'virulence' genes), reporters from these genus/species underrepresented on ASPv5.3 due to lack of completed genome sequences however positive genes give information of bacteria (e.g. antibiotic resistance) and any positive ribosomal genes can be used to predict to the level of family/genus, 5 = None indicates no significant match with any BLAST predictions within current database, 6 = *S. maltophilia *sequence not available at time of analysis, 7 = identification of UU's using ribosomal genes (n/a = not appropriate if species can be elucidated), if present, based on the number of genera which the ribosome is present in at least 1 species (number of predicted genera including duplicates).

#### "Known knowns"

In order to validate the ASP microarray design and the bioinformatic analysis of data, a selection of sequenced isolates were used (table 1). The three examples presented in this study include *F. tularensis *SCHU S4, *S. aureus *COL and *C. difficile *630. BLAST revealed that out of all the 4958 reporters on the microarray, *F. tularensis *SCHU S4 was predicted to hybridize to 27 reporters, *S. aureus *COL 107 (104 chromosomal and 3 plasmid) reporters, whereas *C. difficile *was predicted to hybridize to only two reporters. Hybridisation results were compared to BLAST predictions.

Hybridisation with *F. tularensis *SCHU S4 identified 29 positive reporters (figure 1), consisting of 27 predicted by BLAST. The two additional reporters were Cg34500228 and Cp39102563, however BLAST analysis of these reporters against the *F. tularensis *SHCU S4 genomes did not produce a match of greater than 22/26 bp, which was not predicted to be sufficient for hybridisation.

Hybridisation with *S. aureus *COL DNA confirmed 101 of 107 BLAST predicted reporters in at least one of three hybridisations. Five predicted reporters produced weak signal just below the signal intensity cut-off and one produced negligible signal. Five additional reporters present by hybridisation were not predicted by BLAST. Four genes predicted to be present in *S. aureus *COL by hybridisation and/or BLAST were chosen for analysis by vectorette PCR to determine if the reporters were cross-hybridising to non-specific DNA. Haemolysin (Sa29510762) was used as a proof of principle control, it was positive in both BLAST prediction and hybridisation experiments. Exotoxin 3 (Sa29510468) was predicted by BLAST to be positive, yet the hybridisation failed for this reporter and finally two reporters positive by hybridisation but not predicted by BLAST; bleomycin resistance (Sa29520032) and chloramphenicol resistance (Cv50850700). The vectorette PCR amplified a band for the haemolysin, which was sequenced and proven to be the haemolysin gene (SACOL0762) (table 2). The exotoxin 3 was also confirmed as exotoxin 3 by vectorette PCR (table 2), suggesting the hybridisation conditions used for the arrays were not optimal for this specific reporter. Interestingly, the vectorette PCR for bleomycin and chloramphenicol resistance did not produce products with any of the four different *S. aureus *COL vectorette libraries (table 2), suggesting that the hybridisations on the microarray may have been due to miss-priming, or binding to non-specific DNA.

**Table 2:** Summary of BLAST prediction, hybridisation data and vectorette PCR for putative *S. aureus *COL genes

Gene Name/phenotype	Gene ID	BLAST/hyb	Vectorette product	Top Hit	Size
Haemolysin	SACOL0762	Yes/Yes	Yes	SACOL0762	126/126
Exotoxin 3	SACOL0468	Yes/No	Yes	SACOL0468	362/362
Bleomycin resistance	SAR0032	No/Yes	No	n/a	n/a
Chloramphenicol resistance	CV0700	No/Yes	No	n/a	n/a

*C. difficile *strain 630 was used to test the microarray, there were no reporters specifically designed from *C. difficile *630, as only the pre-release genome sequence was available at the time of design and was therefore not included. *C. difficile *630 CDS information was obtained from the pre-release genome using Artemis  and BLAST analysis against the ASPv5.3 oligonucleotides was performed. Two reporters on the microarray (M50000021 and Sa41160923) had 100% identity to two genes within *C. difficile *630, a duplicated erythromycin resistance transferase (Cd630-2007 & -2010) and a tetracycline resistance gene (Cd630-0508). Hybridisations with *C. difficile *630 genomic DNA revealed three positive reporters, the two mobile genetic elements as predicted, and the type III secretion protein HrpT (Ps45781390) designed from *Pseudomonas syringae *DC3000. By BLAST, Ps45781390 had only a partial match to the *C. difficile *630 genome. The presence of the two predicted genes in *C. difficile *630 was confirmed using vectorette PCR (methods section) using the vectorette libraries derived from *C. difficile *630.

#### "Known unknowns"

Eight clinical isolates were obtained from Kings College Hospital, London and had been identified to species level and tested for antimicrobial susceptibility using conventional methods. However, these pathogens had not been sequenced and therefore BLAST analysis could not be performed, but hybridisations results could be compared to BLAST predictions from other sequenced strains. These pathogens were used to test the ability of the ASP microarray to identify, distinguish and predict gene content. Two bacterial pathogens, *S. aureus *and *E. faecium*, are described here as examples.

The *S. aureus *strain tested on ASPv5.3 was a Panton-Valentine leukocidin (PVL) positive Community Acquired (CA)-MRSA with known antimicrobial resistances (ampicillin^R^, meticillin^R ^and penicillin^R^). Hybridisations identified 112 positive reporters on the ASP microarray which most closely resembles *S. aureus *strain MRSA252 (hypergeometric probability p-value of 5.87e^-129^) (table 1). The positive ASP reporters included four meticillin resistance-associated genes and a β-lactamase precursor, which corroborates the known antimicrobial resistance phenotype. Additionally, putative resistances to bicyclomycin, bleomycin, fluoroquinolones, chloramphenicol, sufamethoxazole, and sulphonamide were identified. Although sensitive to antibacterial glycopeptides, this isolate carries two teicoplanin resistance-associated genes, BLAST analysis of these ASP reporters sequences identified matches with 100% identity in fourteen *S. aureus *complete genomes. Interestingly, both genes required for PVL and an additional leukocidin were also detected, which corroborates the known toxin phenotype. Additionally, a further 13 exotoxins were identified; an exfoliative toxin A, an α-haemolysin, γ-haemolysin (components A, B & C), a putative haemolysin, a putative vacuolating cytotoxin and a β-haemolysin. Two *S. aureus *pathogenicity islands were partially identified, one from the bovine *S. aureus *RF22 (5/7 reporters) and the other from *S. aureus *COL (3/9 reporters). Of interest were 12 non-*S. aureus *reporters, six of which were designed from plasmids (table 3). This indicates that the ASP microarray has identified genes that are potentially novel to *S. aureus*, which may have been acquired by horizontal gene transfer (table 3).

**Table 3:** Potential horizontally acquired genes predicted by ASP microarray in a clinical CA-MRSA isolate

Reporter	Annotation	Information source	Location
Ba693320065	Type IV secretion system protein VirB5	*Brucella abortus*	Chromosome
Bq595505520	Alpha-hemolysin	*Bartonella quintana*	Chromosome
Bq595505860	Invasion associated locus B (IalB) protein family	*Bartonella quintana*	Chromosome
Cv50850766	Probable multidrug resistance protein	*Chromobacterium violaceum*	Chromosome
M49450015	Ethidium bromide resistance protein QacEdelta1	*Corynebacterium glutamicum*	Plasmid
M49450016	Sufamethoxazole resistance protein SulI	*Corynebacterium glutamicum*	Plasmid
M49730008	Sulfonamide-resistant dihydropteroate synthase	Uncultured eubacterium	Plasmid
Nm31162146	*Neisseria*-specific antigen protein, TspA	*Neisseria meningitides*	Chromosome
pFCM1Amp	Ampicillin resistance	Cloning vector (Tn7 based)	Plasmid
pRSB10115Su	Dihydropteroate synthetase type 1 confers sulfonamide resistance	Uncultured eubacterium	Plasmid
pRSB10116Qac	Quaternary ammonium resistance	Uncultured eubacterium	Plasmid

The two vancomycin-resistant *E. faecium *(VRE) isolates, distinguishable by tetracycline^R ^(KCH1) and linezoid^R ^(KCH2), were both resistant to ampicillin, erythromycin, gentamicin, penicillin, rifampicin and trimethoprim. DNA from both isolates were tested in duplicate on the ASP microarray. ASPv5.3 did not contain any specific *E. faecium *reporters, however the microarray did contain 40 reporters from the related species *E. faecalis *(strain V583). As no *E. faecium *strains have been sequenced it was not possible to predict which, if any, *E. faecalis *V583 reporters would hybridise. Hybridisations on the ASP microarray identified eight genes that were present in both strains of VRE, including five vancomycin resistance related genes, four of which matched the vancomycin^R ^operon genes found on the *S. aureus *plasmid pLW043. The other four hybridisation positives were to an erythromycin resistance transferase and two genes from the related organism *E. faecalis*; an adhesion lipoprotein and a S14 ribosomal protein. The ribosomal reporter S14 was used for identification of the bacteria, however the discriminatory power of this particular ribosomal reporter was limited to the phylum firmicutes due to sequence homology from bacteria within two different orders (Bacillales and Lactobacillales). The reporter list for VRE KCH1 contained the tetracycline reporter, which was absent in the reporter list for the second test sample VRE KCH2; thus ASP data corresponds to the known sensitivities of these bacteria as indicated above. BLAST analysis of the tetracycline reporter produced a 100% match to a *E. faecium *tetracycline resistance gene (accession number AY081910). VRE KCH1 ASP hybridisation results also identified potential resistance conferred by streptothricin acetyltransferase and streptomycin aminoglycoside 6-adenyltransferase. However, susceptibility to these agents is not routinely determined. To confirm this *in-vitro*, susceptibility testing was performed using streptomycin on both VRE, each isolate exhibited an MIC of >128 mg/L.

The reporter lists for both of the VRE strains have been incorporated into the gene lists in GeneSpring, which importantly, this will expedite the identification of future VRE isolates.

#### "Unknown unknowns"

*Stenotrophomonas maltophilia*, a member of the γ-proteobacteria, is a pathogen found in immunocompromised patients and is naturally resistant to many broad-spectrum antimicrobials, making it often difficult to treat. Until the very recent publication of the *S. maltophilia *K279a genome sequence in 2008 [17] little genetic information was available for this organism. A clinical isolate of *S. maltophilia *was obtained from the Kings College Hospital with no information on the antimicrobial susceptibility profile. The reporter list identified it as a β- or γ-proteobacteria species by the ribosomal reporter (Bp63503218). Six further reporters were linked to antimicrobial resistance and detoxification (table 4), which may give an insight into the drug resistances of this isolate of *S. maltophilia*. For example reporter (Ps70052483) was annotated as conferring acriflavin resistance and *S. maltophilia *ULA-511 has been demonstrated to be acriflavin resistant [MIC 256 μg/ml] [18]. The subsequent release of the *S. maltophilia *K279a genome, allowed a BLAST analysis to be performed, which confirmed a match of between 43/50 bp to 47/50 bp for five out of seven reporters (table 4). The remaining two reporters were specific to this clinical isolate.

**Table 4:** Positive ASP microarray reporters from a clinical isolate of *S. maltophilia*

Reporter	Annotation	BLAST match with *S. maltophilia *K279a
As65131772	Bacterial type II secretion system protein E	43/50
Bp63503218	30S ribosomal protein S12	45/50
Bt76501375	Heavy metal efflux pump CzcA	43/50
Pf41290909	Superoxide dismutase, Mn	47/50
Ps70052483	Acriflavin resistance protein	45/50
Se69051442	Secretion system apparatus SsaT	no hit
Tt38691142	ABC-type multidrug transport system, permease component	no hit

Five reporters predicted by ASP were also present in the *S. maltophilia *K279a sequence strain but two genes were specific to the clinical isolate

The ASP microarray in this case was able to identify the bacterium to either a β- or γ-proteobacteria class, and importantly provided some insight into the potential antibiotic resistance profile of this understudied organism. This positive reporter list was added into the hybridisations database to expedite the distinction of *S. maltophilia *from other bacteria.

#### Classification of unknown bacteria using ribosomal reporters

Ribosomal reporters were added to the ASP microarray to aid identification of test organisms, however 16S and 23S were not selected due to their high level of conservation among species. The discriminatory power of the ribosomal reporters was assessed in Table 1. For example, a test organism identified eleven ribosomal genes (table 5) on the ASP microarray, these ribosomal reporters were then used in a BLAST analysis, which identified 11 bacterial genera, all predicted by BLAST to contain at least one of the ribosomal reporters (table 5). All of these 11 bacterial genera fell within the γ-proteobacteria. Further analysis revealed that six of the genera are all members of the Enterobacteriaceae family, however, more importantly all eleven reporters are only present in the *Yersiniae *(table 5). Furthermore, one reporter (Yp58100797) appears to be specific to the *Yersiniae *(table 5), therefore using only the ribosomal reporters only, it suggests that the test organism was a member of the γ-proteobacteria and most likely of the genus *Yersinia*.

**Table 5:** Ribosomal reporters identified in an unknown isolate (*Y. pseudotuberculosis*) and their predicted conservation within the prokaryotes

Reporter	*Enterobacter*	*Erwinia*	*Escherichia*	*Haemophilus*	*Pasteurella*	*Photorhabdus*	*Salmonella*	*Shewenella*	*Shigella*	*Yersinia*	*Vibrio*
Family	E	E	E	P	P	E	E	S	E	E	V
Hi09070800				Y	Y		Y			Y	
Vc25052592	Y	Y	Y				Y		Y	Y	Y
Vc25060290								Y		Y	Y
Yp58100052	Y	Y	Y				Y		Y	Y	
Yp58100112										Y	
Yp58100200			Y						Y	Y	
Yp58100206	Y	Y	Y			Y	Y		Y	Y	
Yp58100214		Y								Y	
Yp58100215	Y						Y			Y	
Yp58100233	Y	Y	Y			Y	Y		Y	Y	
Yp58100797										Y	
Total (44)	5	5	5	1	1	2	6	1	5	11	2

E = Enterobacteriaceae, P = Pasteurellaceae, S = Shewenallaaceae, V = Vibrionaceae, Y = reporter identified in at least one species from that genera by BLAST analysis.

#### Discussion

The ASP array has validated over 823 gene sequences as suitable reporters from 19 pathogens. The reporters include both chromosomally located and plasmid encoded antimicrobial resistance cassettes, pathogenicity islands, secretion systems, effectors, toxins, drug export and detoxification systems designed from 99 different genera, 151 species, 205 genomes (additional file 3). In addition, accompanying reporters were designed from ribosomal proteins to aid in genera differentiation. This provides the opportunity to test an unlimited number of bacteria species to determine the content of their potential virulence determinants. One limitation of DNA hybridisations is that the presence of a gene reporter does not demonstrate that the gene is functional. For example a pathogen may carry a drug resistance gene or part of a resistance cassette/operon but may be currently sensitive to that antibiotic, however the presence of a non-expressed resistance gene does indicate that there is a higher potential for resistance to be developed under exposure to the right antibiotics, which could influence choice of treatment antibiotic.

The ASPv5.3 microarray was tested and validated on a range of bacterial pathogens, from those with available genome sequences (e.g. *F. tularensis *SCHU S4, *S. aureus *COL and *C. difficile *630) to those that are clinically important, but have limited information regarding antimicrobial resistance profile or genetic information, such as *S. maltophilia*. The *S. aureus *COL validation of the ASP microarray demonstrated that accurate prediction of positive reporters on the ASP microarray was achievable and that this data, confirmed by vectorette PCR, could be used successfully to identify bacteria with a very high level of certainty. The majority of predicted reporters produced positive signals, including plasmid-based genes, with only a few absent. As many antibiotic resistance genes are plasmid mediated, it is important to ensure extraction of both chromosomal and plasmid DNA for testing. In the case of *S. aureus *COL 3 BLAST predicted genes were located on the *S. aureus *COL plasmid pT181, all of which were positive in a least one hybridisation, suggesting that the 'chromosomal' preparation also extracted plasmid DNA, however the limitation is that the cannot distinguish chromosomal versus plasmid encoded. As predicted there were a few differences between observed (hybridisations) and predicted (BLAST) results, which did not adversely effect the identification of the bacteria. These differences can be attributed to hybridisations or bioinformatics errors. For such a large array, including reporters for a wide range of bacteria it was predicted that some of the reporters would fail, either in design, synthesis, microarray production or under the hybridisation conditions for the oligonucleotides. There are a number of ways of addressing the issue, however all have their limitations, one could increase stringency by increasing the value of the minimum positive signal cut-off to result in fewer false positives, yet this would produce more false negatives. The reducibility of the replicate spots is a critical factor for being defined as positive, greater accuracy could be achieved with either more replicates or multiple reporters per gene. However, duplicate reporters per gene would increase the cost of oligonucleotides, yielding little additional data. Both methods would greatly increase (up to double) the size of the microarray and physical space on the glass slide is finite. Improvements in printing density and microarray fabrication can currently manufacture up to 1,000,000 reporters directly onto a slide for a fixed cost, which would enable a greater number of reporters, and, therefore the physical size of the microarray, but practically this may result in lower signal as the labelled DNA is diluted and dispersed over a greater area. Sensitivity, signal strength and hybridisation time could be further improved by dynamic hybridisation using microfluid devises, rather than the static incubation used in this study. The bioinformatic analytical methodologies will be assessed and improved with the next version of the ASP array, along with an increase in the number of reporters per gene and the number of genes covered from an ever-expanding variety of microorganisms for which genome sequence data is becoming available.

Analysis of *C. difficile *630 hybridisations demonstrated useful genetic information can be obtained even when there were no specific reporters for an organism on the ASP microarray. Two of the positive reporters were designed from drug resistance genes present on mobile elements Tn*916 *in *Streptococcus agalactiae *2603 V/R and the plasmid pRUM in *E. faecium *U37. These genes have been demonstrated to confer resistance to tetracycline and erythromycin in *C. difficile *630 [19], which demonstrates that the ASP microarray can be used to obtain potential antimicrobial resistance information when the genome sequence is unavailable. The third positive reporter (Ps45781390) only had limited similarity with the *C. difficile *630 chromosome, and was most likely cross-hybridizing to either an integrase (weak match (25/50)) match with an integrase or to the *C. difficile *630 plasmid p630 (weak match (27/50), 1 gap). This reporter was most likely cross-hybridising. To improve the data from the ASP microarray for this important nosocomial pathogen, *C. difficile *specific reporters will be added to the next iteration of ASP microarray design.

Analysis of clinical isolates such as VRE and CA-MRSA confirmed that the ASP microarray could provide useful information in the identification process as well as important genetic information, such as pathogenicity traits and antimicrobial resistance, even when no VRE specific reporters were on the ASP microarray. Two clinical isolates of VRE were successfully distinguished through the presence of tetracycline resistance in one strain. The tetracycline reporter matched 100% to a previously described *E. faecium *resistance gene although it was not stated whether the gene was located chromosomally or on a plasmid, however this reporter was also found in vancomycin resistance genes located on two *E. faecalis *plasmids (pAMα1 and pJH1). The information gained from the hybridisation patterns obtained with these two strains improved the potential for correct identification of VRE from future samples, where information on strain or antimicrobial resistance profile is unavailable.

Analysis of the clinical CA-MRSA isolates was successfully identified as *S. aureus*, VPL-positive and meticillin resistant. Additional information revealed putative antimicrobial resistances and toxins previously unknown for these strains. Furthermore eleven genes encoding important drug resistance determinants were identified in the clinical isolate, using reporters from other species. Interestingly, six of which are known to be encoded on mobile elements suggesting horizontal gene transfer.

The genetics of the emerging pathogen *S. maltophilia *have only recently begun to be deciphered. However, the ASP microarray was able to identify seven genes, one ribosomal and six virulence-associated genes in a clinical isolate of *S. maltophilia*. Five out of seven reporters were subsequently confirmed to have matches in the recently published *S. maltophilia *K279a genome sequence [17] including the ribosomal and acriflavin resistance genes.

Sample identification was also proved successful with a blind test was performed on *Y. pseudotuberculosis *strain 0:56 using only information from the ribosomal reporters. These reporters identified *Y. pseudotuberculosis *as a γ-proteobacteria and most likely *Yersinia *(table 3). Additionally, the combination of ribosomal and virulence genes as a whole correctly distinguished this as *Y. pseudotuberculosis *(p = 8.63e-199) from *Y. pestis *(p = 3.44e-188). The ability to differentiate between these close relatives, indicates that the ASP microarray will also be able to differentiate between pathogens and closely related non-pathogenic species.

Inclusion of further antibiotic resistance markers will increase the utility of the ASP microarray for predicting the antibiotic resistance profile of unknown pathogens. As the microarray analysis can be undertaken more rapidly than traditional antibiotic susceptibility assays, this may help in guiding clinicians in their choice of therapy. For some pathogens, prompt initiation of antibiotic therapy in essential. For example, CDC guidelines in the treatment of plague are that antibiotic therapy should be initiated within 24 h of the development of symptoms. Currently it is possible to hybridise and analyse the ASP microarray in under 24 hours however this requires prior pathogen isolation, growth and DNA extraction and with the emergence of multidrug resistant strains of plague [20-22] there is an obvious need to improve the turn around time. Improvements to the ASP microarray, with development of faster microarray technologies and automation along with direct detection from clinical specimens, would make the ASP microarray invaluable in administering appropriate antibiotic prescription.

Microarray-based technology is ideal for multiple gene identification and, therefore suited for rapid pathogen identification particularly antibiotic resistant strains. To this end, Berthnet *et al. *have recently adapted the Affymetrix resequencing GeneChip technology [23] to identify bacterial and viral species from within a complex (clinical) mixture and also identify and confirm the presence of antibiotic resistances. The advantage of resequencing is that it can identify point mutations in specific genes, including those responsible for antibiotic resistance. However the ASP microarray technology is cheaper and can identify a more comprehensive range of detectable genes.

Finally the concept of the ASP microarray design was to monitor the flow of genetic material between bacteria, both as an epidemiological tool and as a rapid screen of emerging pathogens. To achieve this the primary step is to ensure that 'normal' bacteria can be identified, along with expected virulence factors. This proof of principle paper establishes that the ASP microarray can do both, and has also provided data that some of the tested isolates contain unexpected genetic material demonstrating gene flux.

#### Conclusion

This proof of principle paper has demonstrated that the ASP microarray can be used to identify a wide variety of bacteria. More importantly, this system can also identify virulence-associated genes that have potentially been acquired by horizontal gene transfer. This ranges from information that could be relevant clinically (drug resistances and toxins) to the identification of potential genes that may be important in the emergence of virulent pathogens. The ASP microarray has demonstrated great potential and utility for the rapid surveillance of the gene content of a given bacterium, which could have uses clinically, in aiding diagnosis and identifying appropriate therapeutic regimens. The use may extend beyond the clinic, in following transfer of genes and the emergence of pathogens by pathogenicity island acquisition, for example, as observed by the spread of the Edinburgh-Toronto strain of *Burkholderia cepacia *in cystic fibrosis patients [24]. Additionally the ASP microarray can identify novel genes in a strain, which can be investigated using the reporter sequence and vectorette method. The ASP microarray is an adaptable tool, which will be continually modified, optimised and expanded to cover more pathogens including viruses and further virulence determinants, enhancing its impact and making it more generally applicable for diagnostic and research purposes.

#### Abbreviations

ASP: Active surveillance of pathogens; CDS: Coding sequence; MGE: Mobile genetic element; MRSA: Meticillin resistant *Staphylococcus aureus*; PVL: Panton-Valentine leukocidin; VRE: Vancomycin resistant *Enterococcus faecium*.

#### Competing interests

The authors declare that they have no competing interests.

#### Authors' contributions

RS designed, constructed and tested the ASP microarray, designed and optimised bioinformatics, set up GeneSpring database and helped draft the manuscript, LD tested the ASP microarray, carried out all vectorette work and helped draft the manuscript, PO, RT & JW supplied strains and genetic material and provided scientific input, JH assisted with microarray design, AW assisted with design and PERL scripting, BW conceived the microarray and helped draft the manuscript. All authors have read and approved the final manuscript.

## Supplementary Material

Additional file 1 **ASP oligonucleotide reporters** Click here for file

Additional file 2 **List of oligonucleotides used in the vectorette library construction, vectorette PCR and sequencing** Click here for file

Additional file 3 **ASP oligonucleotide reporters were designed from 99 different bacterial genera including 151 different species and 205 separate fully sequenced genomes.** Click here for file
