# Supplementary material for: Development and application of the active surveillance of pathogens microarray to monitor bacterial gene flux
Source: BMC Microbiol. 2008 Oct 9;8:177. doi: 10.1186/1471-2180-8-177 (PMC2607285; doi:10.1186/1471-2180-8-177)
Supplement: Additional file 2 — List of oligonucleotides used in the vectorette library construction, vectorette PCR and sequencing [file 1471-2180-8-177-S2.doc]

| Primer name | Sequence | Gene | Target |
| --- | --- | --- | --- |
| LFD1F (bubble) | AAGGAGAGGACGCTGTCTGTCGAAGGTAAGGAACGGACGAGAGAAGGGAGAG | vectorette Bubble |  |
| LFD1R (bubble) | CTCTCCCTTCTCGAATCGTAACCGTTCGTACGAGAATCGCTGTCCTCTCCTT | vectorette Bubble |  |
| LFD2 | ATGCTTGCCAATGCTAAGCTCTTCCCT | vectorette mismatch region |  |
| pLFD5 (nested vectorette) | TCGCTAAGAGCATGCTTGCCAATGCTAAGC | vectorette mismatch region nested |  |
| pLFD3 | GCCAGCCCCGTCGTCCAAATAGTCGGATAG | *tetM* | *C. difficile* |
| pLFD4 (nested) | TCGTCCAAATAGTCGGATAGATAAAGTACGA | *tetM* | *C. difficile* |
| pLFD6 | CGAACACTAGGGTTGCTCTTGCACACTCAA | M50000021 | *C. difficile* |
| pLFD7 (nested) | GGTTGCTCTTGCACACTCAAGTCTCGATTCA | M50000021 | *C. difficile* |
| pLFD8 | CCCAATACATCATTAAAAGATCAGTGGTGG | S29520032 | *S. aureus* |
| pLFD9(nested) | CATTAAAAGATCAGTGGTGGGATGAACGAGA | S29520032 | *S. aureus* |
| pLFD10 | GCTGGTATTGCTGGCCTTGAGCTTCACCGC | Cv50850700 | *S. aureus* |
| pLFD11(nested) | CTGGCCTTGAGCTTCACCGCCGTATCCAAAT | Cv50850700 | *S. aureus* |
| pLFD12 | CCCAATGTTGTGTTTTGGATTGGGTGGAAT | S29510898 | *S. aureus* |
| pLFD13(nested) | TGTTTTGGATTGGGTGGAATCAAGAAGCGTT | S29510898 | *S. aureus* |
| pLFD14 | CGTGGATACAACTTATGATCGCTGGGTTGT | S29510762 | *S. aureus* |
| pLFD15(nested) | ACTTATGATCGCTGGGTTGTTTCAGAAATCG | S29510762 | *S. aureus* |
| pLFD16 | CGACATTCACTAGTATGGGTAATTTCATAG | S29520748 | *S. aureus* |
| pLFD17(nested) | TAGTATGGGTAATTTCATAGGTCCTTTAATC | S29520748 | *S. aureus* |
| pLFD18 | AGAGAAGGTGACAGTAGACAAGCAACAAAT | S29510468 | *S. aureus* |
| pLFD19(nested) | ACAGTAGACAAGCAACAAATTACTCAATTG | S29510468 | *S. aureus* |
| M13 Forward | GTAAAACGACGGCCAG |  | pGEM |
| M13 Reverse | CAGGAAACAGTATGAC |  | pGEM |
