# Supplementary material for: Development and application of the active surveillance of pathogens microarray to monitor bacterial gene flux
Source: BMC Microbiol. 2008 Oct 9;8:177. doi: 10.1186/1471-2180-8-177 (PMC2607285; doi:10.1186/1471-2180-8-177)
Supplement: Additional file 3 — ASP oligonucleotide reporters were designed from 99 different bacterial genera including 151 different species and 205 separate fully sequenced genomes. [file 1471-2180-8-177-S3.doc]

|  | Genus | Species | Genomes |
| --- | --- | --- | --- |
| 1 | Acinetobacter | 1 | 1 |
| 2 | Aeropyrum | 1 | 1 |
| 3 | Agrobacterium | 1 | 2 |
| 4 | Anaplasma (Ehrlichia) | 1 | 1 |
| 5 | Aquifex | 1 | 1 |
| 6 | Archaeoglobus | 1 | 1 |
| 7 | Azoarcus | 1 | 1 |
| 8 | Bacillus | 7 | 13 |
| 9 | Bacteroides | 2 | 3 |
| 10 | Bartonella | 2 | 2 |
| 11 | Bdellovibrio | 1 | 1 |
| 12 | Bifidobacterium | 1 | 1 |
| 13 | Bordetella | 3 | 3 |
| 14 | Borrelia | 2 | 2 |
| 15 | Bradyrhizobium | 1 | 1 |
| 16 | Brucella | 3 | 3 |
| 17 | Buchnera | 2 | 3 |
| 18 | Burkholderia | 3 | 3 |
| 19 | Campylobacter | 1 | 2 |
| 20 | Candidatus | 3 | 4 |
| 21 | Caulobacter | 1 | 1 |
| 22 | Chlamydophila | 1 | 1 |
| 23 | Chlamydia | 4 | 8 |
| 24 | Chlorobium | 1 | 1 |
| 25 | Chromobacterium | 1 | 1 |
| 26 | Clostridium | 3 | 3 |
| 27 | Colwellia | 1 | 1 |
| 28 | Corynebacterium | 3 | 3 |
| 29 | Coxiella | 1 | 1 |
| 30 | Dehalococcoides | 1 | 1 |
| 31 | Deinococcus | 1 | 1 |
| 32 | Desulfovibrio | 1 | 1 |
| 33 | Ehrlichia | 3 | 5 |
| 34 | Enterococcus | 1 | 1 |
| 35 | Erwinia | 1 | 1 |
| 36 | Escherichia | 1 | 4 |
| 37 | Francisella | 1 | 1 |
| 38 | Fusobacterium | 1 | 1 |
| 39 | Geobacter | 1 | 1 |
| 40 | Gloeobacter | 1 | 1 |
| 41 | Haemophilus | 2 | 3 |
| 42 | Halobacterium | 1 | 1 |
| 43 | Helicobacter | 2 | 3 |
| 44 | Lactococcus | 1 | 1 |
| 45 | Legionella | 1 | 3 |
| 46 | Leifsonia | 1 | 1 |
| 47 | Leptospira | 1 | 2 |
| 48 | Listeria | 2 | 3 |
| 49 | Mannheimia | 1 | 1 |
| 50 | Methanococcus | 1 | 1 |
| 51 | Mesorhizobium | 1 | 1 |
| 52 | Methanopyrus | 1 | 1 |
| 53 | Mesoplasma | 1 | 1 |
| 54 | Methylococcus | 1 | 1 |
| 55 | Methanosarcina | 2 | 2 |
| 56 | Methanothermobacter | 1 | 1 |
| 57 | Mycobacterium | 1 | 1 |
| 58 | Mycoplasma | 6 | 6 |
| 59 | Neisseria | 2 | 3 |
| 60 | Neorickettsia (Ehrlichia) | 1 | 1 |
| 61 | Nitrosomonas | 1 | 1 |
| 62 | Oceanobacillus | 1 | 1 |
| 63 | Phytoplasma | 1 | 1 |
| 64 | Pasteurella | 1 | 1 |
| 65 | Photorhabdus | 1 | 1 |
| 66 | Porphyromonas | 1 | 1 |
| 67 | Prochlorococcus | 1 | 1 |
| 68 | Pseudomonas | 4 | 5 |
| 69 | Psychrobacter | 1 | 1 |
| 70 | Pyrobaculum | 1 | 1 |
| 71 | Pyrococcus | 1 | 1 |
| 72 | Ralstonia | 1 | 1 |
| 73 | Rhodopseudomonas | 1 | 1 |
| 74 | Rickettsia | 4 | 4 |
| 75 | Salmonella | 2 | 6 |
| 76 | Shewanella | 1 | 1 |
| 77 | Shigella | 1 | 2 |
| 78 | Silicibacter | 1 | 1 |
| 79 | Staphylococcus | 4 | 13 |
| 80 | Streptococcus | 5 | 15 |
| 81 | Streptomyces | 1 | 1 |
| 82 | Sulfolobus | 1 | 1 |
| 83 | Synechococcus | 1 | 1 |
| 84 | Synechocystis | 1 | 1 |
| 85 | Thermoanaerobacter | 1 | 1 |
| 86 | Thermosynechococcus | 1 | 1 |
| 87 | Thermotoga | 1 | 1 |
| 88 | Thermus | 1 | 1 |
| 89 | Treponema | 1 | 1 |
| 90 | Tropheryma | 1 | 1 |
| 91 | Ureaplasma | 1 | 1 |
| 92 | Vibrio | 1 | 1 |
| 93 | Wigglesworthia | 1 | 1 |
| 94 | Wolbachia | 1 | 1 |
| 95 | Wolinella | 1 | 1 |
| 96 | Xanthomonas | 1 | 1 |
| 97 | Xylella | 1 | 1 |
| 98 | Yersinia | 2 | 4 |
| 99 | Zymomonas | 1 | 1 |
|  | Total | 151 | 205 |
